# Looking for Creativity: Where Do We Look When We Look for New Ideas?

**DOI:** 10.3389/fpsyg.2016.00161

**Published:** 2016-02-15

**Authors:** Carola Salvi, Edward M. Bowden

**Affiliations:** ^1^Department of Psychology, Northwestern UniversityEvanston, IL, USA; ^2^Rehabilitation Institute of ChicagoChicago, IL, USA; ^3^Department of Psychology, University of Wisconsin–Parkside, Kenosha, WIUSA

**Keywords:** creativity, imagination, eye movements, blink rate, attention, insight problem solving

## Abstract

Recent work using the eye movement monitoring technique has demonstrated that when people are engaged in thought they tend to disengage from the external world by blinking or fixating on an empty portion of the visual field, such as a blank wall, or out the window at the sky. This ‘looking at nothing’ behavior has been observed during thinking that does not explicitly involve visual imagery (mind wandering, insight in problem solving, memory encoding and search) and it is associated with reduced analysis of the external visual environment. Thus, it appears to indicate (and likely facilitate) a shift of attention from external to internal stimuli that benefits creativity and problem solving by reducing the cognitive load and enhancing attention to internally evolving activation. We briefly mention some possible reasons to collect eye movement data in future studies of creativity.

It has been said that the eyes are the windows on the soul. In this paper we will examine whether the eyes are the windows to the mind. We will concentrate on eye movements and blinking, and how they both reveal and influence creative thinking.

Anecdotally, when people are engaged in retrieving information from memory, imagining, problem solving or thinking creatively, they often shift their gaze from the problem or from other people toward an empty space or a blank wall. This was popularly understood to be a way to disengage from distracting information so that one can concentrate on inner thoughts. The artist Paul Gauguin described his necessity of disengaging from outside reality to enhance his imagination and creativity in his famous quote ‘I shut my eyes in order to see.’

The connection between thinking and visual processes has a long history in psychology, predating the development of eye tracking equipment and neuroimaging techniques. One of the founders of psychology as a science, William James (1890), remarked on the connection between eye movements and cognitive processes in his ‘Principles of Psychology’:

When I try to remember or reflect, the (eye) movements in question, instead of being directed toward the periphery, seem to come from the periphery inward and feel like a sort of *withdrawal* from the outer world. As far as I can detect, these feelings are due to an actual rolling outward and upward of the eyeballs, such as I believe occurs in me in sleep, and is the exact opposite of their action in fixating a physical thing. (http://psychclassics.yorku.ca/James/Principles/prin10.htm)

Clearly as early as 1890, when ‘Principles of Psychology’ was first published, psychologists had noticed a possible relation between eye movements and cognition. During the first half of the 20th Century, Gestalt Psychologists suggested that there are many processes that are shared by visual perception and problem solving. For example, perceptual organization that determinates various aspects of vision, such as object recognition and depth perception have analogs (if not the same processes) in problem solving that lead to both difficulties and to sudden insights. One of the key points of the Gestalt principles of visual perception is that object recognition can come suddenly and holistically, following a reorganization of the visual elements into a new integrated whole. Analogously, while engaged in a creative activity, or in problem solving, a new idea or a new solution can arise suddenly and holistically from a reinterpretation or reorganization of the problem elements. Gestalt psychology faded with the cognitive revolution in the 1950s, largely due to its theories being more descriptive than explanatory (Bruce et al., 1996), yet the similarities, at least at a surface level, between visual and problem solving processes had been established. In the second half of the 20th Century non-Gestalt perception psychologists, most notably Rock (1983), suggested that much of visual perception is, in fact, a form of intelligent problem solving rather than merely the result of automatic processes.

More recently, the Embodied Cognition movement has led to increased interest in how cognitive processes and seemingly non-cognitive body processes are linked. Embodied cognition is the belief that cognitive processes and the body are not separate but are linked at the most basic level. The connection between thought and the body is bidirectional with thought influencing and directing the states and actions of the body (e.g., Lakoff and Johnson, 1980, 1999), and states and actions of the body influencing and directing thought (e.g., Eerland et al., 2011).

Two fundamentals assumptions underlie the position we take in this paper: The first is the evolutionary position that existing structures and systems are not replaced by new ones, rather that new ones are built on top of the existing ones (Jonides et al., 2005), the second is that the connection between thought and the body is bidirectional.

## Eye Movements and Memory

Existing neural systems that enable search for information in the visual environment may have given rise to neural systems able to search for non-visual information stored in long-term memory (Ehrlichman and Micic, 2012). The connection is supported by the finding that when people search for (non-visual) information in long-term memory they make multiple eye movements analogous to those made when searching the environment visually, and when they focus on information in long-term memory they make very few eye movements just as happens when people focus on an object in the visual environment. Thus, mechanisms for internal attention may have evolved from those already in place for attending to the external world. In this theory Ehrlichman and Micic (2012) speculate that internal thought processes are systematically related to (non-visual) eye movements. More frequent movements of the eyes are found when people are engaged in tasks that require search of long-term memory than when they are engaged in tasks that do not require long-term memory search, even when the tasks do not seem to have any visual component. In fact, Ehrlichman and Weinberger (1978) found that participants were less likely to make eye movements (were more likely to stare) when answering visuospatial questions than when answering verbal questions. Bergstrom and Hiscock (1988) found that differences in eye movements are related to the memory demands of questions, and Glenberg et al. (1998), found that when people try to respond to difficult questions they avert their gaze from engaging visual inputs.

## Looking at Nothing or at Objects that Aren’T there

Hebb (1968) introduced the idea that imagining an object is associated with the same eye-movement scan paths that would be associated with actually viewing that object. He suggested that eye movement during imagery has a functional role, to, as in perception, put together and organize the ‘part images’ to construct a complete visualized image. More recent empirical evidence demonstrated that eye movements during imagery are not random, but reflect the content of the imagined scene (Brandt and Stark, 1997) therefore imagining any visual stimulus triggers corresponding oculomotor responses as if thinking of an object involves pretending to look at it. In a series of studies, Spivey and Geng (2001) demonstrated how eye movements mirror mental images. In two experiments (Spivey and Geng, 2001) participants were instructed to look at a white projection screen while (1) imagining (by listening to pre-recorded instructions of specific directionality, i.e., rightward, leftward, upward, and downward) or (2) recalling objects that were not physically present on the display in what was called the Hollywood squares paradigm; (Richardson and Spivey, 2000), they were asked to recall a characteristic of one of the four objects that was previously presented on the screen). Results of the first experiment showed that participant’s eye movements were biased toward the direction of the spatiotemporal imagery in the scene description. Results of the second experiment showed that participants were more likely to look at the blank region of the screen where the missing object had been presented, despite the fact that looking at the blank region was not informative because there was no visual information there to address the recall. The results have been replicated even when participants were asked to relax and to close their eyes while listening to the ten short stories (i.e., there were no instructions to specifically imagine anything) (Spivey et al., 2000).

Several cognitive-neuroscience models suggest memory retrieval is based on the recreation of cortical processes that were active at the time of the original experience (e.g., Marr, 1971; Norman and O’Reilly, 2003). Indeed, it has been shown that common neural systems are activated during initial perception and later retrieval (e.g., Nyberg et al., 2000; Wheeler et al., 2000). Brandt and Stark (1997) demonstrated that during recall of picture or scenes participants’ eyes moved spontaneously and the movements closely reflected the spatial relations and the overall content of the imagined picture or scene. Johansson et al. (2006) further investigated this phenomenon by comparing eye movements in four conditions: (1) while participants listened to a spoken scene description and (2) when participants were later retelling it from memory; (3) while studying a complex picture visually, and (4) when they were later describing it from memory. When participants were instructed to recall a visual scene, previously presented either via a verbal description or visual scene, the eye movements made during initial listening or viewing spontaneously reappeared with recall of the scene. This repetition of the eye movements made at encoding occurred for participants both when they looked at a blank white board and in conditions of complete darkness (see also Ehrlichman and Barrett, 1983). Thus, remembering a visual scene seems to involve the reinstatement of the visual processes that were active during encoding. Johansson and Johansson (2014) hypothesized that the probability of remembering should improve when the visual processes engaged at retrieval overlap with those engaged at encoding. Their results demonstrated that spontaneously looking at a blank area and positioning the eyes on a location congruent with the location of stimuli during encoding facilitates retrieval. In fact, when participants made different eye movements at the time of recall than they had made at encoding they showed a decrease in their ability to recall the visual scene (Laeng and Teodorescu, 2002).

It is important to note that participants in the verbal description condition (Johansson et al., 2006) never saw a visual stimulus, yet the overlap between eye movements at the time of recall with those made at the time of encoding still predicted recall performance. Why would this be so? Ferreira et al. (2008) examined this ‘looking at nothing’ behavior, and suggested that it reflects an integrated memory representation based on visual and linguistic input. They proposed that reactivation of one part of the representation results in the other parts being retrieved as well. Thus, there is a feedback loop in which, for example, the reactivation of a linguistic component of the memory can cause the eyes to move to the location in which the item originally appeared, and that returning the eyes to this location can improve memory for other information associated with that item, including any visual and conceptual information. Looking at the location where a stimulus was presented, even after it is no longer present, is a result of the integrated representation, and facilitates retrieval of further information from it.

It seems reasonable that if looking at a location that was previously occupied by a visual stimulus can aid in the recall of both visual and conceptual information, then looking *away* from a still present stimulus, or from the place the stimulus previously occupied, could serve the opposite purpose. That is, looking at nothing could reduce the tendency to perseverate on an idea, or reduce that information’s level of activation and its ability to capture and maintain attention. According to Ferreira et al. (2008) this would apply to both the visual elements of the stimulus and any associated conceptual information. This also suggests that longer fixations away from a stimulus would predict better inhibition of retrieval and less interference, whereas shorter fixations away and frequent returns to the stimulus would predict poorer inhibition and greater interference.

## Eye Movements and Attention

Some of the clearest evidence for the bidirectional connection between eye movements and cognition comes from studies of attention; the direction of a person’s gaze is generally a clear (though not perfect) indication of where attention is directed. When a person wants to attend to an object or spatial location she/he moves her/his eyes so that she can fixate on the object or location, and when a person’s eyes moves to an object or location attention usually moves too.

Attention is central to perceptual and higher level cognitive processes, and it plays a central role in creativity (Kounios and Beeman, 2009, 2014). Though attention and eye movement are not one and the same, they are tightly linked (Golberg and Wurtz, 1972; Posner, 1980; Rizzolatti et al., 1987; Klein et al., 1992; Hoffman and Subramaniam, 1995; Kowler et al., 1995; Deubel and Schneider, 1996). The pre-motor theory of attention (Rizzolatti et al., 1987) suggests a strict link between both overt and covert orienting of attention and programming explicit ocular movements. Given a limited capacity to process competing external stimuli, attention selects, regulates, and maintains focus on the information relevant for behavior while simultaneously inhibiting processing of information that is also available, but that is irrelevant. It seems likely that control of attention has evolved out of the necessity to efficiently manage limited cognitive processing capacity and focus it on the information most relevant to ongoing goals and behaviors (Pashler et al., 2001). Attention guides and controls how we move our eyes, allowing us to handle the wealth of visual information provided by the surrounding environment (Rayner, 2009; Schall and Thompson, 1999). Multiple stimuli compete for selection, and the goal of attention is to bias the competition to favor a target object (Desimone and Duncan, 1995). Processing of a target object would be facilitated by both enhancing activation of the target and inhibiting distractors and noise. A very straightforward way to inhibit processing of an irrelevant stimulus would be to look away from it, close one’s eyes, or engage in increased blinking.

The pre-motor theory of attention (Rizzolatti et al., 1987) suggests that eye movements and attentional shifts are driven by the same internal mechanisms, and they are both managed mostly by the superior colliculus (Golberg and Wurtz, 1972).

Chun et al. (2011), propose a taxonomy based on the types of information that attention operates over (i.e., the target of attention): Information coming in from the outside, through the senses (external or bottom-up attention), and information that already has an internal representation (internal or top–down attention). External attention is driven by properties of the outside world and involves the selection and modulation of sensory information, in a modality-specific representation, and often with tags for spatial locations and time (Chun et al., 2011). These two types of attention share the same capacity limitations and they are mutually exclusive. In other words, if attention to internal thoughts is increased, attention to the external world will decrease, and vice versa.

Several studies, using different behavioral and neurophysiological measures and across different fields, e.g., problem solving (Jung-Beeman et al., 2004; Kounios et al., 2006; Kounios and Beeman, 2009; Salvi et al., 2015), mind wandering (Smallwood et al., 2007; Schooler et al., 2011; Smallwood et al., 2011, 2013), have produced converging evidence demonstrating that, when attention is focused internally processing of external stimuli is suppressed. This mechanism would allow the enhancement of internal concentration by reducing distractions.

As mentioned above, studies of gaze aversion have shown that the frequency of ‘looking away’ increases with the difficulty of cognitive processing, that it has a functional consequence on memorization, and that closing the eyes improves accuracy for questions of moderate difficulty (Glenberg et al., 1998). Analysis of eye blink rates has shown a similar pattern. Blinking physically blocks incoming information by the closing the eyelid, and generates a suppression of vision associated with an inhibitory signal sent out by the brain (Volkmann et al., 1980) both before and after the time of actual lid closure (Stevenson et al., 1986; Volkmann, 1986; Bristow et al., 2005a,b). But, blinking is something more than a mere interruption of visual input, it has been suggested that blinking is a sensory ending of a top-down processes that allows or facilitates an internal and more complex cognitive mechanism of attention (Salvi, 2013). Specifically, directing attention internally has been found to produce higher eye blink rates (Wood and Hassett, 1983). According to Holland and Tarlow (1975), blinking occurs at the moment of cognitive change as an indicant of transitions between different gazes, sets, or ideas. Conversely, both blink rate and blink duration decline as a function of more intense mental workload (Brookings et al., 1996; Hankins and Wilson, 1998; Veltman and Gaillard, 1998), task concentration, mental activity, and when information in memory is being operated on (Telford and Thompson, 1933)–such as solving arithmetic problems (Holland and Tarlow, 1975). Blinking has been consistently found to be associated with internal thought processes like insight problem solving (Salvi et al., 2015), creativity and divergent thinking (Akbari Chermahini and Hommel, 2010; Ueda et al., 2015), mind wandering (Smilek et al., 2010), errors in vigilance related to external stimuli (Van Orden et al., 2000; Papadelis et al., 2007), and conflicts between internal and external workloads (Recarte et al., 2008).

## Eye Movements, Blinking, and Mind Wandering

Our attention fluctuates over time between being internally and externally focused (Smallwood et al., 2008a,b). Mind wandering is a state of focus on internal information, where our attention switches from the primary task to our private thoughts that become the focus of awareness (Smallwood and Schooler, 2006). This entails a state of processing decoupled from perception, and a temporary failure in meta-awareness, i.e., the ability to self- reflect upon the content a mental state (Schooler, 2002).

When looking for a creative idea, or the possible solution to a problem, people often mind wander. Of course, mind wandering does not necessarily imply that one is working on a problem or having a creative idea, for example one could be thinking of a past vacation. However, several lines of evidence converge to show that mind wandering is related to both creativity (e.g., Baird et al., 2012; Ritter and Dijksterhuis, 2014) and the ‘looking at nothing’ behavior mentioned before (Smilek et al., 2010).

Mind wandering has been associated with both internally focused attention and with reduced cortical analysis of the external environment (Smallwood et al., 2008a). Specifically, when people try to engage attention in a sustained manner the depth of cognitive analysis applied to the external environment fluctuates. Reichle et al. (2010) established that a person’s fixation pattern while reading is a reliable indicant of attentive or mindless reading. Their results demonstrated that right before episodes of mind wandering subjects were more likely to avoid the text, looking somewhere else, and to elongate their fixations. This study, demonstrates that mind wandering competes with the processing of task-relevant information and reduces the cognitive analysis of external events (e.g., Dehaene and Changeux, 2005; Smallwood and Schooler, 2006). Smilek et al. (2010) showed that an increased blinking rate is associated with mindless reading. Their study demonstrated that mind wandering is coupled with physical blocking of sensory information provided by closing the eyes, suggesting that eye blinks can serve as an index of the degree to which a person is attending to internal thoughts. Thus, the evidence from the variety of studies discussed above supports the position that eye movements and blinking can be used to infer cognitive processes such as memory search and focus of attention.

## Eye Movements, Blinking, and Creativity

Problem solving often implies the construction of mental models (Johnson-Laird, 1983). Mental models serve to both depict the abstract relations between objects and events, and orient attention toward information relevant for the mental model (Smallwood et al., 2008b). Demarais and Cohen (1998) showed that when reasoning, specifically during syllogisms containing the words ‘left’ and ‘right’, participants make more horizontal eye movements, and during syllogisms containing ‘above’ and ‘below’ participants make more vertical eye movements. Similarly, when attempting to solve problems we often imagine the abstract representation of the problem space and the elements that make up the problem scenario. The creation of models can be triggered in an automatic bottom–up manner (Gerrig, 2005) or constructed in a more purposeful analytic, top–down manner (Graesser et al., 1994), with most problem solving efforts involving an interaction between the two.

Another way to conceptualize problem solving is to suggest that people solve problems via heuristic search through a problem space (Newell and Simon, 1972). In this space the various pieces of information given in a problem and other previous knowledge, which may have initially seemed unrelated, can become linked together to reach a solution. One way to reach a solution is to search the problem space by analysis, following the most likely paths in a gradual approach toward solution with awareness of the intervening steps (Metcalfe and Wiebe, 1987). Alternatively, a novel solution to a problem can suddenly emerge into consciousness in what is called *Insight* or an ‘Aha!’ moment. Insight is a form of creative problem solving that appears to be distinct from analytic solutions because it relies on the sudden reorganization of a mental representation of a problem (Sternberg and Davidson, 1995), and it often seems surprising to the solvers, who are typically unaware of how the reorganization occurred, yet remain confident that the solution fits the whole problem.

Cognitive neuroscience has begun to shed light on specific components that underlie problem solving in general, and the differences between creative and more analytic problem solving styles. Neuroimaging reveals that, within a network of neural substrates engaged during problem solving, distinct areas are recruited or emphasized when people solve with sudden insight compared to when they solve analytically (for review: Kounios and Beeman, 2014). Much of this evidence at least circumstantially suggests that distinct patterns of attention (broad or narrow focus) differentiate the two types of solutions. We suggest that evidence from eye movement recordings adds significantly to this understanding.

The association between eye movements and thinking has been demonstrated for some time. Early studies suggested that the direction of eye movements indicates increased activation of the contralateral cerebral hemisphere. Bakan (1969) found that leftward eye movements in response to questions were associated with clearer mental imagery and relatively poorer mathematical performance on the Scholastic Aptitude Test (SAT). Harnad (1972) found that among twenty participants the ten who predominantly moved their eyes in a leftward direction had higher RAT (Mednick and Mednick, 1967) scores and made more extreme esthetic ratings than the right-movers. Kocel et al. (1972) found that the direction of lateral eye movements was strongly modified by the type of question, with verbal and arithmetical questions eliciting more rightward eye movements than did spatial and musical questions. Each of these studies suggests at least a weak relationship between the tendency to look in one direction and the level of performance on certain tasks that would appear to require either more analytic or more creative thinking. Hines and Martindale (1974) went a step further and examined whether artificially induced right or left eye movements facilitated intellectual and creative tasks, respectively. In two experiments male left-lookers scored significantly higher than male right-lookers on the RAT and Alternate Uses Test (AUT; Guilford, 1967). However, in a third experiment female right-lookers scored non-significantly higher than female left-lookers on the RAT and AUT. Hines and Martindale concluded that the effect of induced lateral eye movements was real but relatively weak.

Recent research by Shobe et al. (2009) and Fleck and Braun (2015) has continued to support the effect of lateral eye movements on creativity. Shobe et al. (2009) investigated the effects of increased inter-hemispheric interaction on five dimensions of creativity (appropriateness, detail, flexibility, fluency, and originality) of the AUT. They used a bilateral eye movement task to directly manipulated inter-hemispheric interaction. They found that bilateral eye movements increased originality and flexibility of participants with a strongly dominant hand, but had no effect on mixed-handers. Fleck and Braun (2015) combined the visual-hemifield presentation technique with eye movement tasks to investigate whether the bilateral eye movement effect could be extended to a creativity task similar to the RAT [the Compound Remote Associates (CRA) Task; Bowden and Jung-Beeman, 2003]. They found that eye movement conditions resulted in improved performance on a solution-recognition task, with the bilateral eye movement condition demonstrating the best performance for solution targets.

Studies using the visual-hemifield presentation technique have provided more specific data on how the hemispheres contribute differentially to creativity. Two studies showed that participants were faster to read, and recognize as solutions, solution words presented to the left visual field, and thus initially the Right Hemisphere (Bowden and Beeman, 1998; Jung-Beeman and Bowden, 2000). This indicated that there was greater activation of solution-relevant information in the right hemisphere than in the left hemisphere. A further visual-hemifield study showed that following unsolved problems participants showed greater priming for solutions that they rated as evoking an insight experience on the subsequent solution decision than for solutions that did not evoke an insight experience. This association was stronger for solutions presented to the left visual field-RH than for those presented to the right visual field-LH. These results tied the subjective experience of insight to an objective measure—semantic priming—and suggested that people have an *Aha!* experience in part because they already had semantic activation that could lead them to recognize the solution quickly (Bowden and Jung-Beeman, 2003). The evidence suggests that semantic activation in both hemispheres cooperatively contributes to problem solving, whereas weak solution activation that contributes to the *Aha!* experience is more likely to occur in the RH than in the LH. These studies were followed up by a neuroimaging study combining fMRI and EEG, which revealed increased activity in the right hemisphere anterior superior temporal gyrus for insight solutions relative to non-insight solutions (Jung-Beeman et al., 2004). This area is associated with making connections across distantly related information during comprehension (Beeman et al., 2000). Thus, what was hinted at by early studies of the relation between lateral eye movements and activation of the hemispheres was ultimately fine tuned to show that specific areas within each hemisphere play important and distinct roles in problem solving.

Eye movements have also been used to test theories of problem solving. For example, Knoblich et al. (2001) used recording of eye movements to test the representational change theory of insight. They predicted that when a person is at an impasse that person should have fewer eye movements (longer fixations) and that fixation patterns would reveal what parts of the problem people consider relevant, so that the elements of the problem that people fixated on would change following constraint relaxation. They found that for successful problem solvers, the percentage of fixation time spent on the element that must be changed to reach a solution increased over time and that the percentage of fixation time increased dramatically in the time period immediately prior to solution. Their study demonstrates the power of eye movement recordings to reveal facets of problem solving that would have remained hidden when using more traditional performance measures like solution time and solution rate.

Bilalić et al. (2008) were able use eye movement data to reveal the mechanisms of Einstellung (cognitive set). They demonstrated that once chess experts had found one solution they continued to look at the squares related to their first idea, even while reporting that they were looking for a better idea. In other words, the fixations showed that chess experts were continuing to allocate most of their attention to their first idea even when they thought they were searching for other possible moves. Ellis et al. (2011) and Ellis and Reingold (2014), further investigated Einstellung by monitoring participants’ eye movements while they attempted to solve anagrams. The anagrams were presented as six letters: a central three-letter string whose letters were always part of the solution word, and three additional individual letters one of which was a distractor letter (not part of the solution word). Participants were asked to find a five-letter solution word. The central letter string was presented as either a nonword or as a three-letter word. The typical Einstellung effect was found with better overall performance for nonword than word trials, however, participants’ eye movements revealed both interference and facilitation as a function of the familiarity of the central letter string. Participants spent less time looking at the central letters when they were presented as a word, suggesting that a word was easier to encode and maintain in memory a word than three individual letters. Participants also spent more time viewing the individual letters when the central letters were presented as a word, suggesting that they found it more difficult to incorporate the individual letters into an existing central word.

Not only can eye movements reveal underlying processes and strategies, they can influence these processes and strategies. Based on Grant and Spivey’s (2003) suggestion that there is an implicit link between eye movements and cognition, Thomas and Lleras (2009a) subtly guided the eye movements of some participants as they attempted to solve Duncker’s (1945) radiation problem (Duncker, 1945)–*Given a human being with an inoperable stomach tumor, and lasers which destroy organic tissue at sufficient intensity, how can one cure the person with these lasers and, at the same time, avoid harming the healthy tissue that surrounds the tumor?*^1^ Thomas and Lleras (2009a) found that using an eye-tracking task to induce eye movements that embodied the solution led to an increase in solution rates compared to participants who were allowed to move their eyes freely. In another study Thomas and Lleras (2009b), required participants to either move their eyes, move their attention while holding their eyes still, or keep both their eyes and attention fixated in the center of the display. The results show that shifting attention in a pattern that expressed the problem’s solution increased the probability of solving the problem even in the absence eye movements. Therefore, it is not the eye movements themselves that lead to the solution; rather it is the effect the eye movements have on attention that is important.

Thomas (2013) also manipulated eye movements while participants attempted to solve a problem, and simultaneously performed a verbal or spatial working memory task. Participants who moved their eyes in a pattern that embodied the solution were more likely to solve the problem than participants who maintained fixation, however, this was only true for participants who performed the verbal working memory task. Since the solution to the radiation problem relies largely on spatial information, loading spatial working memory prevented the eye movement manipulation from improving problem solving. In contrast, when paired with the verbal memory task the eye movement manipulation improved solving performance just as in the earlier studies.

Of course, to influence solution rates by directing eye movements it would seem that one has to know what the solution is. However, Werner and Raab (2014, p. 1572) advance the argument that ‘it is not the movement *per se* but the goal of the movement that is causal for the effect of sensorimotor information on cognitive processes’ (Raab and Green, 2005; Engel et al., 2013). Therefore, influencing eye movements, and blink rates, might also work at a level more general or abstract than the specific solution by influencing attention and memory retrieval. Eye movements to, and fixations on, a stimulus can serve to focus attention on that external stimuli and facilitate the retrieval of further information from it, whereas eye movements away from the stimulus, and fixations on empty space, can serve to shift attention away from external stimuli, inhibiting further processing, and allowing weaker (more distantly related) internal information to come to the fore.

In a study using CRA problems Jung-Beeman et al. (2004) strongly discouraged (so as not to get eye movement artifacts in EEG data) this tendency to look away or blink. They found that EEG revealed a sudden increase in alpha-frequency activity over the right occipital-parietal cortex 1.5 s prior to insight solutions, but not prior to analytic solutions. Alpha activity over sensory cortex is thought to indicate active suppression of input (Haegens et al., 2011; Händel et al., 2011; Ben-Simon et al., 2013) so this increase in alpha under the condition of restriction in eye movements and blinking was interpreted as a covert effort to reduce the amount of visual information passed from visual areas to higher areas that perform more abstract computation (Kounios and Beeman, 2009; Payne and Kounios, 2009). In other words, this alpha burst was interpreted as attention shifting away from the visual representation of the problem, toward internal processing (Benedek et al., 2014) just prior to insight but not solutions by analysis.

Results from an fMRI study of problem solving indicate that during the rest period prior to presentation of visual problems stronger activity in dorsal Anterior Cingulate Cortex (dACC) is associated with subsequently solving by insight, whereas stronger activity in visual cortex is associated with subsequently solving by analysis (Kounios et al., 2006). The ACC is a critical hub in the network subserving cognitive control (Posner and DiGirolamo, 1998; Kerns et al., 2004), the process by which the brain guides and controls processing, and plays a pivotal role in attention shifting (Kondo et al., 2004). The greater neural activity found over the visual cortex–prior to problems solved analytically-suggested that participants are pre-oriented to elaborate visual information thus more prone to direct their attention outwardly (Kounios et al., 2006; Kounios and Beeman, 2009). This perspective is consistent with the more general idea that creative thinkers have the ability to change cognitive states between defocused and focused attention (Martindale, 1995), strategically inhibit peripheral information when necessary (Stavridou and Furnham, 1996), and allocate attention in a diffuse manner (Dykes and McGhie, 1976). Wood and Hassett (1983) have shown that internally directed attention yields higher blink rates during problem solving and Tsubota et al. (1999) demonstrated that the visual cortex activation is greater with voluntary blink inhibition. Thus, solving by insight is associated with directing attention toward cognitive control – perhaps enhancing the ability to shift processing from one idea to another – whereas solving by analysis is associated with a readiness to process visual information. This latter conclusion has been demonstrated by measuring eye movements when people have insights. In a study paralleling the neuroimaging and EEG work, Salvi et al. (2015) monitored overt attention, before and while people solved word problems, through three different measures: blinking, fixation frequency, and fixation location. The results suggest that solutions via insight are facilitated by actively reducing potentially interfering visual inputs, by blinking more before and during problem solving, and avoiding visual distractors by looking in the white space outside of the problem area just prior to solving. These results directly demonstrate that people overtly direct their attention differently when solving a set of CRA problems by insight versus solving by analysis. Decreased blinking and increased number of fixations show that externally focused attention, on the problem itself or the space where the problem will appear, is more conducive to solving problems with analysis. Increased blinking and decreased eye movements, as well as increased fixations on empty space, indicate that internally focused attention on associations generated by the problem, is more conducive to solving with sudden insight. This pattern of blinking, fixations, and eye movements–which indicate disengaging from the visual stimuli–appear to enhance imagination and creativity by diminishing processing of inputs and their strong associations, and switching attention inwardly to allow detection of weaker associations.

Ueda et al. (2015) also found that blinking could have an effect on creativity. Analyses of eye blinks during creative performance indicated that increased eye blink rates corresponded with the production of more alternative uses on the AUT and slower solutions on the RAT. They suggest that the slower RAT solutions reflect a more divergent search for solution candidates, an interpretation that would be consistent with increased eye blinks being related to using an insight approach to solve RAT problems. The results are compatible with the suggestion that spontaneous eye blinks are actively involved in attentional disengagement from the external world allowing more divergent thinking to occur.

## A Third Variable Interpretation of Eye-Movements, Blinking, and Creativity

As we have discussed above, there is a fair amount of evidence that eye movements and blinking reflect basic cognitive processes such as memory search (e.g., Ehrlichman and Weinberger, 1978; Ehrlichman and Barrett, 1983; Bergstrom and Hiscock, 1988; Glenberg et al., 1998; Laeng and Teodorescu, 2002; Ferreira et al., 2008; Ehrlichman and Micic, 2012; Johansson and Johansson, 2014) and attention (e.g., Golberg and Wurtz, 1972; Posner, 1980; Rizzolatti et al., 1987; Klein et al., 1992; Hoffman and Subramaniam, 1995; Kowler et al., 1995; Deubel and Schneider, 1996; Salvi et al., 2015), and can be used to infer these cognitive processes with a reasonable degree of accuracy (Henderson et al., 2013). There is also evidence (though less) that eye movements and blinking can affect, as well as reveal, cognitive processes (e.g., Shobe et al., 2009; Thomas and Lleras, 2009a,b; Thomas, 2013; Fleck and Braun, 2015). However, a third possibility that we have not discussed is that eye movements and blinking, and cognitive processes such as divergent thinking and ability to focus attention are all affected by a third variable. One possible candidate for the third variable is a person’s level of dopamine.

Spontaneous blink rates have been linked to dopamine function (DA; Karson, 1983; Blin et al., 1990; Kleven and Koek, 1996; Taylor et al., 1999; Colzato et al., 2007, 2009) and spontaneous eye blink rate is used as an index of striatal dopamine production (Karson, 1983; Shukla, 1985; Taylor et al., 1999). DA, in turn, is highly associated with executive function, and anatomically with the dACC that contributes to these processes. Increasing dopamine levels or activity via lesions or pharmacology, increases blink rates; conversely, decreasing dopamine decreases blink rates (Karson, 1983; Akbari Chermahini and Hommel, 2010).

Dopamine functioning is associated with attention/cognitive control and with the degree to which people maintain ongoing processes or switch to new processes (Müller et al., 2007). This kind of cognitive flexibility, important for generating new ideas, depends on dopamine D2 receptor signaling (Van Holstein et al., 2011).

Specifically, there are a number of reasons to believe that dopamine plays an important role in creativity. Eysenck (1993) has related aspects of creativity to schizophrenia, and pointed out that schizophrenics and healthy creative individuals share a certain lack of constraints and inhibition in their thinking. Ashby et al. (1999) attempting to explain the beneficial effect of mood on creative behavior assume that higher DA levels are associated with less inhibition of alternative thoughts and greater cognitive flexibility (cf., Cohen and Servan-Schreiber, 1992).

Akbari Chermahini and Hommel (2010) measured EBR in the resting periods before and after participants performed both a divergent thinking task (the Alternative Uses Task – of Guilford, 1967) and convergent thinking one (the Remote Associate Task – RAT, Mednick, 1962). For the AUT, participants who produce moderate levels of spontaneous eye blinks perform better than those with higher or lower levels. Conversely, the researchers found a weak, negative relationship between EBR and RAT accuracy. Akbari Chermahini and Hommel (2010) provided evidence regarding the relationship between creativity and spontaneous eye blinks at rest and their results strengthen the claim that creativity and the dopamine system are related. Thus, both EBRs and convergent and divergent thinking might be the result of different levels of dopamine.

One difficulty with reaching conclusions regarding the relation between dopamine and creativity by using the AUT and RAT (or CRAs) is that the AUT is a relatively pure measure of divergent thinking (with four components originality, fluency, flexibly, and elaboration) while the RAT requires a combination of divergent and convergent thinking. Both the RAT and CRA require that solvers generate many possible words that are related (or form compounds with) the words in the triads, which is a divergent component of the task. Solvers must then be sensitive to the overlap between the many words generated, which is a convergent component of the task, to be able to select the one correct answer. Studies have often focused only on solution rates, used few problems, and given relatively long solution periods, possibly obscuring some subtle changes in EBR or eye movement and fixation patterns.

As mentioned above, Ueda et al. (2015) also found that blinking could have an effect on creativity. They found essentially the same relation between ERB and AUT as reported by Akbari Chermahini and Hommel (2010), however, they examined solution speed as well as solution rate for RAT problems and found that participants with higher resting EBRs took longer to solve RAT problems. They suggest that the slower RAT solutions reflect a more divergent search for solution candidates. They also found no correlation between number solved and EBR during the task suggesting at least the possibility that solution rate is too coarse a measure to be used in studies of the components of creative problem solving.

Salvi et al. (2015) divided solutions to the CRAs into those produced via insight or via analysis (as self-reported by participants) and found that the number of blinks was greater for problems solved with insight than for problems solved with analysis. These results demonstrate that people overtly direct their attention differently when solving a set of CRA problems by insight versus solving by analysis.

Dopamine level has also been found to correlate with creativity in a neuroimaging study (de Manzano et al., 2010). de Manzano et al. (2010) found that scores on divergent thinking tests (Inventiveness battery, Berliner Intelligenz Struktur Test) were correlated with regional D2 receptor densities, as measured by Positron Emission Tomography. The results showed a negative correlation between D2 density in the thalamus and divergent thinking scores, demonstrating that the D2 receptor system is important for creative performance.

Several researchers have examined the emergence of artistic or creative behaviors in people with Parkinson’s Disease (PD) who are undergoing treatment with levodopa or dopamine agonists (Canesi et al., 2012; Inzelberg, 2013).

By examining creative and non-creative PD patients Lhommée et al. (2014) found that creativity decreased significantly following reduction in dopaminergic treatment leading to the conclusion that creativity is at least partly dependent on dopamine. High dopamine levels may disrupt latent inhibition (which is the capacity of the brain to filter seemingly irrelevant stimuli from conscious awareness; Chakravarty, 2010) via alterations in the mesolimbic and mesocortical dopaminergic pathways, which are involved in the modulation of reward, motivation, inhibitory control, and decision-making (Kulisevsky et al., 2009). Reduced latent inhibition is thought to be a biological basis of creativity, therefore, the general hypothesis is that dopaminergic drugs may reduce inhibitory control through the stimulation of these pathways (Antonini and Cilia, 2009), possibly leading to greater creativity.

It is worth mentioning that PD is associated with some problems in visual processing and ocular-motor control (Armstrong, 2011). Some of these symptoms, such as hypometria of saccades, which is related to poor visual memory (Hodgson et al., 1999; Shaunak et al., 1999), are related to dopamine deficiency, while others such as visual hallucinations and prolonged latency of saccades are related to treatment with dopamine agonists (Michell et al., 2006).

## Conclusion

Where do we look when we look for a creative idea? The evidence suggests that we ‘look nowhere’ and we blink. That is, looking at nothing, or the ‘blank wall behavior’ and increased blinking, has been found to be associated with insight, but not analytic solutions on RAT and CRA problems, and increased creativity on various measures of creativity such as the AUT, and better performance on mental imagery tasks. This looking nowhere behavior, as well as increases in blink rate, has also been observed during memory encoding and search, changes in attentional focus, and mind wandering–all forms of cognition that do not explicitly involve visual imagery. The research has demonstrated that ‘looking at nothing’ and blinking are associated with reduced analysis of the external visual environment. Thus, looking nowhere appears to indicate (and likely facilitate) a shift of attention from external to internal stimuli, which benefits creativity and problem solving by reducing the cognitive load and enhancing attention to internally evolving activation.

In this paper we have made a case for using eye movement patterns, fixations, and blink rates to study creativity and problem solving. We suggest that these data can reveal strategies people employ, and how memory search and allocation of attention differ for different types of problem solving. We also suggest that manipulating eye movements might induce attentional states that are more conducive to either analytic or insight problem solving. Eye movements can also be used to reveal what stage a person is at in the problem solving process. For example, eye movements could reveal when a person has reached an impasse and would be most receptive to information that could lead to a change in the representation of the problem, or when a period of incubation might be most effective in the problem solving process.

Finally, we would argue that even if eye movements, blink rates, and fixations are epiphenomena of creativity, or driven by differences in dopamine levels, the fact that they are correlated with different types of cognitive processes suggests that monitoring them during problem solving activities would still provide very useful information.

## Author Contributions

All authors listed, have made substantial, direct, and intellectual contribution to the work, and approved it for publication.

## Conflict of Interest Statement

The authors declare that the research was conducted in the absence of any commercial or financial relationships that could be construed as a potential conflict of interest.

## Footnotes

1 The correct solution to this problem involves firing multiple low-intensity lasers from different locations around the tumor so that they converge at the tumor.
